# Trends in Mortality from Ischemic Heart Disease in Peru, 2005 to 2017

**DOI:** 10.3390/ijerph19127047

**Published:** 2022-06-09

**Authors:** Jesús Austin Vázquez-Troche, Vanessa García-Fernández, Akram Hernández-Vásquez, Rodrigo Vargas-Fernández, Guido Bendezu-Quispe

**Affiliations:** 1 Facultad de Ciencias de la Salud, Universidad Científica del Sur, Lima 15067, Peru; austinvazquez356@gmail.com (J.A.V.-T.); vanessa.garcia.fdz@gmail.com (V.G.-F.); jvargasf@cientifica.edu.pe (R.V.-F.); 2 Centro de Excelencia en Investigaciones Económicas y Sociales en Salud, Vicerrectorado de Investigación, Universidad San Ignacio de Loyola, Lima 15024, Peru; 3 Centro de Investigación Epidemiológica en Salud Global, Universidad Privada Norbert Wiener, Lima 15046, Peru; guidobq@gmail.com

**Keywords:** ischemic heart disease, trends, mortality, international classification of diseases, Peru

## Abstract

The highest proportion of deaths among patients with cardiovascular diseases is due to ischemic heart disease (IHD), which is the second most common cause of death in Peru. This study aims to measure and identify changes in the temporal trend in mortality from ischemic heart disease in the Peruvian population. An ecological study was carried out with data from individual death records from the Ministry of Health between 2005 and 2017. A death was considered attributable to IHD if it was registered with the codes I20 to I25 of the ICD-10. Crude and adjusted mortality rates for IHD were calculated for the general population by age and according to sex. A joinpoint regression analysis was performed to assess trends in IHD mortality. There were 61,524 deaths due to IHD (55.69% men) from 2005 to 2017. According to the ICD-10, acute myocardial infarction (I21) accounted for the highest proportion of deaths (88.16%), followed by chronic IHD (I25), with 6.53%. In general, a decrease in adjusted IHD mortality rates was found in the general population over time (45.34 in 2005; 22.18 in 2017). By sex, men possessed a 1.5-fold higher rate than women. The highest mortality rates from IHD were found in the natural coastal region (68.55%) and in urban areas (86.43%). A joinpoint regression analysis showed a reduction in the mortality trend over time due to IHD for both the general population and the population when grouped by sex. In conclusion, there was a continuous decrease in mortality rates due to IHD in the Peruvian population between 2005 and 2017. Strategies focused on mitigating the impact of this disease are required and should emphasize the subgroups most likely to die from this cause.

## 1. Introduction

Cardiovascular diseases (CVDs) represent the leading causes of death around the world [1], accounting for 18.6 million deaths in 2019 [2]. In addition, ischemic heart disease (IHD) and stroke cause the highest proportion of deaths among patients with CVDs [3], with more than nine million and more than six million deaths occurring in 2019, respectively, with a greater number of deaths occurring in low- and middle-income regions. On the contrary, in high-income regions, a decrease in the mortality rate from IHD has been observed [4,5].

According to the reports by the Global Burden of Disease, IHD is the main cause of mortality in the Latin American region [5,6]. In countries such as Argentina (−51%) and Colombia (−6.5%) the mortality rate decreased between 1985 and 2015, while in Mexico (+61%) it continued to increase during the same period [7]. Specifically, IHD mortality rates in Argentina and Colombia decreased from 137.6 to 67.3 deaths and from 160.6 to 150.7 deaths between 1985 and 2015, respectively, while deaths in Mexico increased by more than two-fold (from 86.5 to 140.8 deaths) during the same period [7]. In addition to Mexico, the specific mortality rate due to IHD in Ecuador (another Latin American country) increased from 74.05 to 84.41 deaths per 100,000 inhabitants from 2001 to 2016 [8]. The factors related to the increase in mortality in low- and middle-income countries include inadequate health infrastructure, poor access to health services and education, and negative changes in lifestyle [5,9,10,11].

The second most common cause of death in Peru is IHD, which has increased by 29.9% in 2019 compared to 2009 and ranks second only to lower respiratory tract infections [12]. Despite the large number of deaths due to IHD in the country, there are few studies on the trends of IHD mortality in the Peruvian population. Consequently, studies are needed to identify changes in mortality trends at both the population level and in population subgroups. Therefore, the objective of this study was to estimate and analyze temporal changes in IHD mortality in Peru in order to identify the populations and regions most susceptible to IHD mortality and develop strategies aimed at mitigating the burden of IHD in Peru.

## 2. Materials and Methods

### 2.1. Study Design and Data Sources

A retrospective, ecological, and serial cross-sectional study was carried out on IHD mortality in Peru between 2005 and 2017.

In 2017, Peru had a population density of 31 million people. The territorial area of the country is 1,285,215.60 km^2^ and it borders Ecuador, Colombia, Brazil, Bolivia, and Chile. According to its geographical characteristics, Peru is divided into three regions: the Coast, on the shores of the Pacific Ocean, which includes the most developed urban centers (including Lima, the capital); the Andes, which has the highest altitudes in the country as it is within the Andes mountain range, and has the population with the lowest level of wealth in Peru; and the Amazon, with its Amazonian biodiversity, which has a population with limited access to health services due to its low geographic accessibility [13]. The highest population density is found in the natural coastal region (which has the most socioeconomic development), followed by the Andes region and the Amazon, where poverty prevails in rural areas. The population growth in Peru is higher in urban than in rural areas, with the presence of internal migration from rural to urban areas [14].

A database of individual death records from the Ministry of Health of Peru (MINSA) was used as a secondary data source. This database included death certificate reports (primary data source) for the entire country, which, among other information, included the basic, intermediate, and final causes of death using the 10th revision of the International Classification of Diseases (ICD-10) codes. It can be accessed by request addressed to the General Office of Statistics and Informatics of the MINSA on the following website: https://www.minsa.gob.pe/portada/transparencia/solicitud (accessed on 14 March 2022).

### 2.2. Variables

A variable for IHD mortality was generated (yes/no). The following death diagnoses for the basic cause of death according to ICD-10 for IHD were included as a yes: angina pectoris (I20), acute myocardial infarction (I21), subsequent myocardial infarction (I22), certain current complications following acute myocardial infarction (I23), other acute IHD (I24), and chronic IHD (I25). The inclusion of these codes was based on their use in previous studies on ischemic disease [4,7,15]. Additionally, data on sex, age, the administrative region of residence, urban or rural area of residence, the district of residence, and the geographic domain of residence (coast/Andean/Amazon) at the time of death were obtained. The determination of the districts as urban or rural was carried out based on the Supreme Decree 090 2011 of the Peruvian government, which contains the list of rural district municipalities in Peru [16].

### 2.3. Statistical Analysis

The methodology described in a study on mortality due to gastrointestinal diseases attributed to alcohol was followed [17]. Initially, the proportion of deaths due to IHD was obtained according to sex (male and female), age group (15–19, 20–24, 25–29, 30–34, 35–39, 40–44, 45–49, 50–54, 55–59, 60–64, 65–69, 70–74, 75–79, and >80), area of residence (urban and rural), and geographic domain of residence (Coast, Andes, and Amazon), as well as the mean and median ages of the deceased. Crude mortality rates for the total population and specific mortality rates according to sex were calculated for each year of the study. Likewise, standardized mortality rates were estimated by age for the total population, by sex, and by administrative region of origin. To calculate age-adjusted mortality rates, the direct method was used with the World Health Organization population (WHO) standard [18], and the rates were plotted on a distribution map for the latest available year. Mortality rates were calculated on the basis of 100,000 people. The information on the number of inhabitants for each year of the study was obtained from the National Institute of Statistics and Informatics of Peru (https://m.inei.gob.pe/estadisticas/indice-tematico/poblacion-y-vivienda/, accessed on 14 March 2022). No correction was applied for the underreporting of deaths.

The temporal trend in mortality from IHD was analyzed with a joinpoint regression model using the Joinpoint Trend Analysis software v4.8.0.1 (*Surveillance* Research Program of the US National Cancer Institute, Bethesda, MD, USA). The Grid Search method was used to locate the joinpoints with a maximum number of 2 and the Bayesian information criterion was also used to determine the optimal model. If significant changes in the mortality trend were identified, the annual percentage change (APC) for the identified study period was reported. A *p* < 0.05 value was considered statistically significant.

### 2.4. Ethical Considerations

The Institutional Research Ethics Committee of the Universidad Científica del Sur (CIEI-CIENTÍFICA, Lima, Peru) approved the execution of the study under the IRB approval no. 040-2020-PRE15. This study served as a partial requirement for two of the authors (J.A.V.-T. and V.G.-F.) to obtain their medical degrees.

## 3. Results

In the period from 2005 to 2017, the total number of deaths due to IHD was 61,524, of which 34,265 (55.69%) were men (Table 1). The mean age of the deceased was 74.5 ± 16.9 years old (men: 71.7 ± 17.1; women: 78.0 ± 15.9) with a median of 78 (interquartile range (IQR): 65–87). Regarding age groups, adults over 65 years old experienced the highest proportions of IHD mortality (Table 1). With respect to the place of residence, the highest proportion of deaths took place in urban areas (86.43%). According to the geographical area, more than half of the deaths due to IHD occurred on the coast (68.55%), with the lowest number being in the Amazon region (6.15%) (Table 1).

Regarding the diagnoses of death according to the ICD-10, acute myocardial infarction (I21) represented the highest proportion of deaths due to IHD in the study period with 88.16%, followed by chronic IHD (I25) with 6.53%. Certain current complications after acute myocardial infarction (I23) accounted for the lowest number of deaths (0.08%) (Table 1). In relation to the years of the study, there was a general decrease in the adjusted IHD mortality rates for the general population during the study period, with the highest age-adjusted IHD mortality rates occurring in the first five-year period evaluated (highest rate in 2005: 45.34) and the lowest in 2015 (18.04) (Table 2). In both men and women, similar patterns to those found for the general population were observed. It was found that men experienced higher adjusted IHD mortality rates than those experienced by women (2017: 26.75 in men and 18.34 in women) (Table 2).

**Figure 1:**
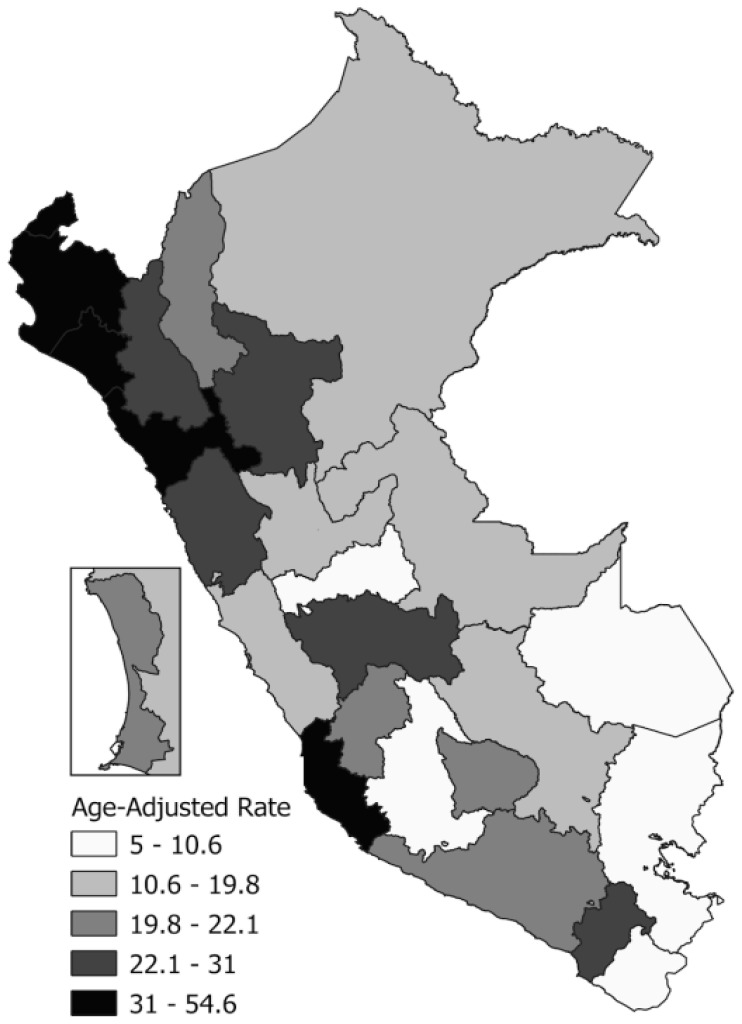
Spatial distribution map for ischemic heart disease mortality rates (per 100,000) in Peru, 2017. To calculate age-adjusted mortality rates, the direct method was used with the World Health Organization population standard [18].

The IHD mortality results, according to the administrative regions of Peru, showed a decrease in the age-adjusted IHD mortality rates during the study period (Table 3). In 2017, coastal regions such as Lambayeque (54.6), Tumbes (50.4), Piura (44.9), and Ica (39.8) represented the highest age-adjusted IHD mortality rates. On the other hand, the administrative Andean regions represented the lowest rates (Figure 1).

**Figure 2:**
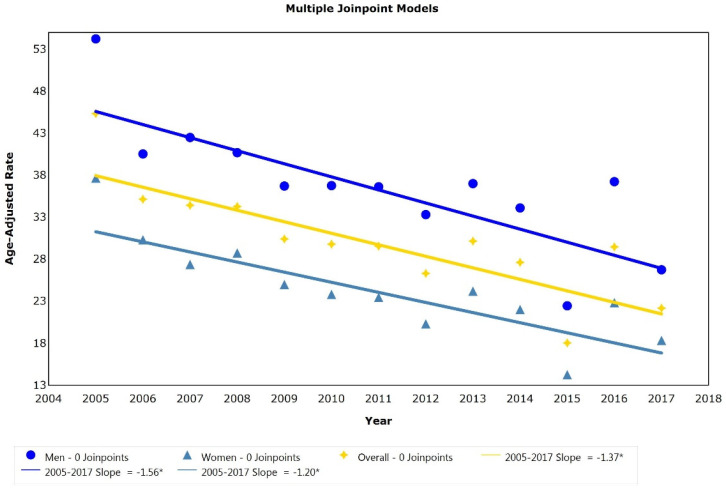
Joinpoint analysis for ischemic heart disease mortality rates adjusted for age (per 100,000) in Peru from 2005 to 2017. * *p* < 0.05. A trend analysis was performed with a joinpoint regression model using Joinpoint Trend Analysis software v4.8.0.1. The Grid Search method was used as well as the Bayesian information criterion to determine the optimal model. To calculate age-adjusted mortality rates, the direct method was used with the World Health Organization population standard [18].

Regarding the results of the joinpoint regression analysis for the Peruvian population, no inflection points were found in the mortality trend due to IHD, with there being a single statistically significant period of decrease in the mortality rate due to this cause (APC: −1.37, *p* < 0.05) (Figure 2). For both sexes, a continuous statistically significant decrease in mortality was also identified during the study period (men: APC: −1.56, *p* < 0.05; women: APC: −1.20, *p* < 0.05) (Figure 2).

## 4. Discussion

This study presents an analysis of the trends in IHD mortality rates in the general Peruvian population and according to sex. The main finding was a decrease in mortality rates due to IHD between the years 2005 and 2017 after adjustment for the age of the general population of Peru and for both sexes. Likewise, higher proportions of deaths from IHD were identified in urban areas (86.43%) and in the natural coastal region (68.55%). In terms of geographic area, the highest IHD mortality rates were observed in Lambayeque (54.6 per 100,000 inhabitants), Tumbes (50.4 per 100,000 inhabitants), Piura (44.9 per 100,000 inhabitants), and Ica (39.8 per 100,000 inhabitants), which belong to the coastal region of Peru. Finally, in the trend analysis, no inflection points were identified in the trend of the adjusted IHD mortality rates during the study period, and therefore, there was only a continuous period of significant decrease in these rates for the general population and both sexes.

The reduction in mortality due to IHD in Peru is related to the global panorama of a decrease in IHD mortality [4,19]. In general, in the Latin American and Caribbean regions, a decrease in deaths from IHD has been described in recent decades [20,21,22]. However, in countries such as Ecuador, Costa Rica, and Mexico, there has been an increase in deaths due to IHD in recent years, which could be explained by the increase in the number of overweight or obese people [7,8,20]. Despite the decrease in deaths from IHD described in Peru, death due to this cause ranks third for the burden of disease (8.2% of the total healthy years of life lost, of which 54.8% were years lost due to premature death) [23]. IHD ranks as the second most common cause of death, only surpassed by lower respiratory diseases [12]. These results make it necessary to continue with the efforts to develop strategies to mitigate IHD in the population, especially for low- and middle-income countries, in which health systems with limited resources must bear a double burden of disease in their population.

The coastal departments showed the highest mortality rates from IHD. Similarly, previous studies have described a higher mortality due to CVDs, including IHD, in this region, and the presence of factors related to CVD mortality in this population [24]. Likewise, higher mortality due to CVDs was found in the urban areas of residence. However, reports in the literature have described that CVD mortality in low- and middle-income countries is higher in rural areas due to significant differences in the educational level and access to quality health services in a timely manner, while in high-income countries, mortality rates are similar in rural and urban areas, which is attributed to universal health coverage that guarantees care in rural and urban areas [25]. The findings of our study could be explained by the fact that the large cities of Peru are in the coastal region, with the population of this region having a higher prevalence of factors related to CVD development or mortality from this cause [26].

A decrease in adjusted IHD mortality rates was found for both sexes, although during the study period, IHD mortality rates were higher for men compared to those of women (approximately 1.5 times) for all the years studied. This result is consistent with the international [4,19] and Latin American regional panorama [20,21,22], which reports a higher mortality due to IHD in men. The literature indicates that men have a higher prevalence of factors related to the development of and mortality due to IHD or CVDs in general, as well as a lower probability of receiving the appropriate treatment for the disease [27,28]. In Peru, men possessed a higher mortality rate due to CVD [24,29] as well as a higher prevalence of factors related to the development of IHD, such as arterial hypertension, compared to women [26]. Likewise, a lower proportion of male patients with CVD showed adequate compliance with treatment [26]. These conditions would increase the probability of Peruvian men presenting with an IHD or dying from this cause. Thus, the development of strategies to reduce IHD and mortality from this cause should take gender into account for the development of specific strategies to mitigate the impact of this disease in the Peruvian population.

Regarding the limitations of this study, it should be specified that as it is a secondary analysis of a database, the accuracy of the data cannot be guaranteed. Therefore, there could have been problems related to the quality of the registration of the death certificates, including the registration of ICD-10 codes and the possible under-registration of deaths. Despite these limitations, the use of a population-level administrative database from the official records of the MINSA constitutes an adequate source of data for the study of trends in mortality due to IHD in the general Peruvian population. In the Peruvian territory (including the most remote districts of Peru’s departments), deaths have been registered manually on a death certificate form, in which the personal information of the deceased, the causes of death, and other relevant information for government institutions are recorded. In addition, the MINSA implemented the “Manual para el llenado del formulario de defunción” in 2009 to contribute to proper completion of these certificates and produce complete and quality vital statistics [30]. This type of registration was carried out until 2016, at which time the MINSA approved the Administrative Directive N°216-MINSA/OGTI-V.01 indicating that death certificates would be registered online through the National Computerized System of Deaths, mainly in districts with adequate internet access. This contributed to the proper identification of the deceased, validation of the professionals who recorded the death, interoperability of information, follow-up of the death registration, and reliability of the data recorded. However, manual death certificate registration continues to be the main form of registration in the most remote districts of Peru [31]. These strategies, which have remained on the agenda of government institutions, provide the greater reliability of the results reported in this study.

## 5. Conclusions

In conclusion, a decrease in mortality from IHD was found in Peru between 2005 and 2017. Subgroups of the population, such as residents of the natural coastal region and urban areas, as well as men, show higher adjusted mortality rates from IHD. Given that IHD continues to lead the causes of death in Peru, it is necessary to develop and evaluate strategies aimed at mitigating the impact of this disease in the Peruvian population, with emphasis on the subgroups with the highest burden of mortality.

## Figures and Tables

**Table 1:** Characteristics of the study population.

Characteristics	Value *n* (%)
Sex	
Male	34,265 (55.69)
Female	27,259 (44.31)
Age (median)	78 (65–87 *)
Age (mean), SD	74.5 (16.9)
Male	71.7 (17.1)
Female	78 (15.9)
Age group (years old)	
15–19	271 (0.44)
20–24	431 (0.70)
25–29	563 (0.92)
30–34	707 (1.15)
35–39	951 (1.55)
40–44	1184 (1.92)
45–49	1637 (2.66)
50–54	2256 (3.67)
55–59	2786 (4.53)
60–64	3725 (6.05)
65–69	4637 (7.54)
70–74	5927 (9.63)
75–79	7596 (12.35)
>80	28,853 (46.9)
Area of residence	
Urban	53,176 (86.43)
Rural	8348 (13.57)
Geographic domain	
Coast	42,174 (68.55)
Andes	15,567 (25.30)
Amazon	3783 (6.15)
ICD-10 causes	
I20	168 (0.27)
I21	54,238 (88.16)
I22	1089 (1.77)
I23	48 (0.08)
I24	1961 (3.19)
I25	4020 (6.53)

* Median and interquartile range.

**Table 2:** Mortality rates (per 100,000) attributed to ischemic heart disease in the study population by sex in the period 2005–2017.

Year	Total Population				Men				Women			
	Crude	Adjusted	95% CI	SE	Crude	Adjusted	95% CI	SE	Crude	Adjusted	95% CI	SE
2005	29.94	45.34	44.15–46.54	0.61	32.89	54.24	52.29–56.19	0.99	27.01	37.66	36.20–39.12	0.74
2006	23.90	35.15	34.13–36.18	0.52	25.51	40.54	38.90–42.18	0.84	22.31	30.32	29.04–31.60	0.65
2007	23.86	34.43	33.44–35.42	0.51	27.28	42.50	40.85–44.15	0.84	20.47	27.38	26.18–28.58	0.61
2008	24.36	34.28	33.31–35.25	0.50	26.50	40.69	39.11–42.27	0.81	22.23	28.75	27.56–29.95	0.61
2009	22.10	30.42	29.52–31.31	0.46	24.48	36.72	35.24–38.19	0.75	19.73	24.99	23.89–26.08	0.56
2010	22.11	29.80	28.93–30.67	0.44	24.97	36.77	35.33–38.22	0.74	19.27	23.82	22.78–24.87	0.53
2011	22.54	29.56	28.71–30.40	0.43	25.65	36.63	35.22–38.04	0.72	19.46	23.46	22.44–24.47	0.52
2012	22.45	26.32	25.54–27.11	0.40	23.7	33.32	32.00–34.63	0.67	17.22	20.31	19.38–21.23	0.47
2013	23.86	30.16	29.33–30.98	0.42	26.73	37.01	35.64–38.37	0.7	21.02	24.20	23.21–25.19	0.50
2014	22.35	27.63	26.86–28.40	0.39	25.15	34.11	32.83–35.40	0.65	19.59	22.02	21.09–22.94	0.47
2015	14.92	18.04	17.43–18.66	0.31	16.88	22.46	21.44–23.49	0.52	12.97	14.26	13.53–14.99	0.37
2016	24.87	29.47	28.70–30.24	0.39	28.55	37.24	35.95–38.53	0.66	21.23	22.83	21.92–23.76	0.46
2017	19.09	22.18	21.53–22.84	0.33	20.79	26.75	25.68–27.83	0.55	17.42	18.34	17.54–19.14	0.41

**Table 3:** Adjusted mortality rates (per 100,000) attributed to ischemic heart disease according to the administrative regions of Peru.

Region	2005	2006	2007	2008	2009	2010	2011	2012	2013	2014	2015	2016	2017
Amazonas	64.1	24.6	29.6	42.2	27.0	9.6	29.7	32.3	18.7	13.0	10.8	15.7	20.6
Ancash	30.7	44.3	36.9	33.9	24.3	25.8	21.4	14.5	18.2	19.8	16.1	14.5	24.6
Apurimac	36.4	36.9	19.6	19.4	11.4	16.3	9.2	12.3	14.2	17.3	8.2	12.0	20.8
Arequipa	47.6	44.9	48.1	42.3	35.6	30.3	25.1	25.0	37.3	41.4	26.6	34.4	20.4
Ayacucho	28.9	29.3	16.9	19.3	19.7	18.9	15.3	18.0	12.0	14.4	10.8	15.6	5.0
Cajamarca	51.1	44.7	48.6	34.4	39.8	24.1	29.5	29.5	24.2	17.5	18.0	15.8	25.6
Callao	45.0	18.7	21.7	26.7	31.0	31.9	31.8	23.4	38.0	31.6	17.6	45.9	21.4
Cusco	40.3	36.7	17.4	16.6	26.4	22.1	12.4	11.5	17.7	12.6	6.6	11.0	12.5
Huancavelica	30.5	19.1	31.2	19.6	21.6	15.5	36.6	27.2	19.9	16.5	16.2	17.0	22.1
Huánuco	59.0	33.3	35.9	47.0	43.9	31.8	27.6	25.7	24.6	25.5	21.3	27.7	14.4
Ica	45.3	46.8	62.7	44.9	41.7	47.3	45.9	38.2	42.4	29.2	23.5	46.2	39.8
Junín	48.0	38.8	25.6	26.7	18.8	21.5	19.3	19.1	10.5	18.6	12.8	11.1	27.3
La Libertad	47.3	33.4	40.3	44.7	45.1	44.7	42.1	32.4	33.2	31.1	24.3	53.9	47.6
Lambayeque	46.2	35.1	37.1	37.6	27.9	36.3	37.4	36.6	45.0	40.4	32.7	48.8	54.6
Lima	46.0	30.3	32.9	33.0	28.3	28.4	26.7	27.2	34.1	29.4	17.1	25.7	13.6
Loreto	10.0	20.6	11.8	16.8	13.5	8.6	13.1	8.2	5.3	1.5	2.7	24.8	10.9
Madre de Dios	10.9	6.5	25.1	19.0	7.0	49.3	11.1	2.0	19.6	49.0	19.3	27.2	9.6
Moquegua	33.3	28.8	21.5	22.6	14.6	13.3	22.5	13.2	9.8	25.6	5.5	13.9	22.2
Pasco	35.7	23.5	31.1	27.1	27.4	39.9	32.1	30.4	23.8	21.4	13.6	18.2	8.9
Piura	64.9	63.1	57.3	67.0	50.7	52.9	72.1	52.7	51.2	50.3	33.5	50.9	44.9
Puno	47.2	27.5	20.3	19.8	21.1	16.8	20.3	15.1	22.4	22.1	11.2	16.7	6.8
San Martin	32.2	29.4	20.4	24.3	20.2	21.1	13.5	21.1	16.4	15.7	8.1	31.9	28.8
Tacna	59.7	54.7	19.8	32.4	29.7	45.3	24.8	17.1	19.6	16.9	13.7	21.7	5.2
Tumbes	57.8	65.6	80.5	64.3	63.9	76.9	58.0	35.8	18.0	21.9	10.1	33.5	50.4
Ucayali	41.4	34.4	48.4	42.4	39.4	26.3	44.5	18.4	17.0	17.5	10.6	2.5	19.0

To calculate age-adjusted mortality rates, the direct method was used with the World Health Organization population standard [18].

## Data Availability

Publicly available datasets were analyzed in this study. These data can be accessed by request addressed to the General Office of Statistics and Informatics of the MINSA on the following website: https://www.minsa.gob.pe/portada/transparencia/solicitud (accessed on 14 March 2022).
